# Debate of Percutaneous Coronary Intervention versus Coronary Artery Bypass Grafting in a Multimorbidity Patient with Complex Coronary Lesions

**DOI:** 10.1155/2020/9493519

**Published:** 2020-05-18

**Authors:** Sotirios Mitsiadis, Nikolaos Miaris, Antonios Dimopoulos, Anastasios Theodosis-Georgilas, Spyridon Tsiamis, Nikolaos Patsourakos, Nikolaos Papakonstantinou, Evangelos Pisimisis

**Affiliations:** Department of Cardiology, “Tzaneio” General Hospital of Piraeus, Piraeus, Greece

## Abstract

**Background:**

While complete revascularization in coronary artery disease is of high priority, the method of implementation in patients with complex coronary lesions and multiple comorbidities is not directed by published guidelines. *Case Presentation*. A 53-year-old female with a chronic total occlusion of the right coronary artery and a bifurcation lesion of the left anterior descending artery and the first diagonal branch, presented with non-ST elevation myocardial infarction. Her past medical history concerned thymectomy and prior chest radiation for thymoma, myasthenia gravis, peripheral artery disease, and cervical cancer treated with surgery and radiation. Although SYNTAX score II favored surgical revascularization, the interventional pathway was finally successfully followed. However, it was complicated with vessel perforation and tamponade managed with pericardiocentesis.

**Conclusion:**

Comorbidities are not all involved in common risk models and require individualization until more evidence comes to light.

## 1. Introduction

Complete revascularization (CR) in patients with multivessel coronary artery disease (CAD) is a crucial prognostic factor that should be taken into consideration in the decision between percutaneous coronary intervention (PCI) and coronary artery bypass grafting (CABG), as it is associated with reduced cardiovascular mortality regardless of the method of implementation [1, 2]. Frequently, the best reperfusion strategy has to be considered with prudence, as CABG in patients with comorbidities may be accompanied by an increased risk for adverse outcomes, which is also high when performing PCI in very complex lesions along with a lower success rate in those cases [3, 4]. The aim of this paper is to present the case of a female patient with two-vessel CAD and such lesion complexity that created skepticism over interventional management, while on the other hand, her simultaneous comorbidities were actually discouraging the surgical option.

## 2. Case Presentation

A 53-year-old woman presented to our emergency department with non-ST elevation myocardial infarction (NSTEMI). She was a 30-pack-year smoker and dyslipidemic on ezetimibe. Her past medical history involved peripheral artery disease (PAD), cervical cancer treated with surgery and radiotherapy 9 years before admission, myasthenia gravis (MG) currently on steroids and pyridostigmine, and thymoma managed with thymectomy (complicated with a myasthenic crisis) and radiotherapy one year before admission.

Four months preceding her current admission, the patient had been hospitalized with non-ST elevation acute coronary syndrome (NSTEACS) and diagnosed with two-vessel CAD that involved a bifurcation lesion extending from the left anterior descending (LAD) artery to the first diagonal branch (D1) and a proximal total occlusion of the right coronary artery (RCA). Lesion complexity indicated reperfusion with CABG, which was then rejected by the patient, and optimal medical treatment was finally adopted.

**Figure 1:**
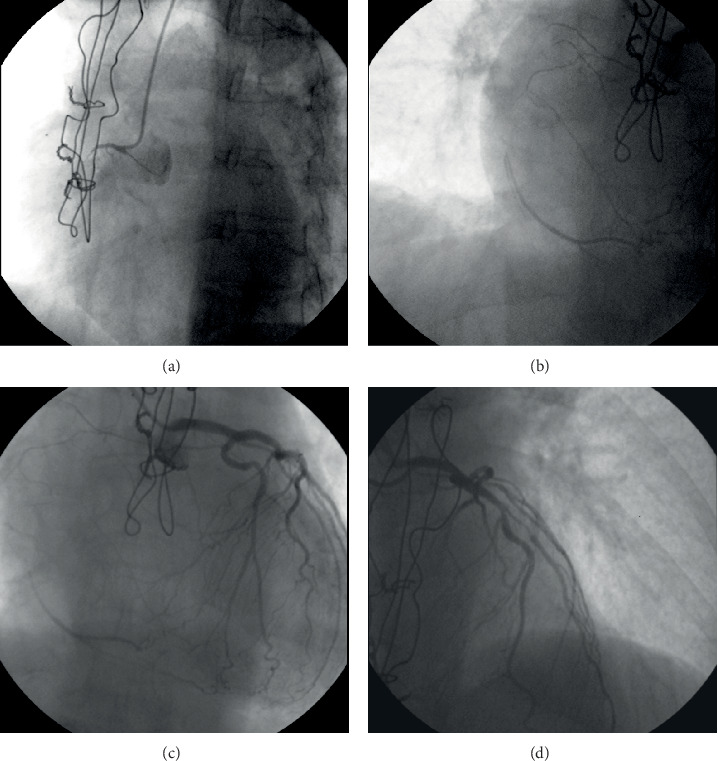
Coronary angiography of the patient. (a) CTO of RCA. (b, c) Collaterals from LAD to RCA territory. Retrograde filling of RCA. (d) LAD/D1 bifurcation lesion.

On admission, the patient presented with chest pain, dynamic electrocardiography changes in multiple precordial leads, and elevated serum cardiac troponin I levels (maximum level of 250 ng/L, cutoff level of 15 ng/L), while mild left ventricular dysfunction (ejection fraction of 45-50%) and inferior wall hypokinesis were revealed by echocardiography. A new invasive coronary angiography (ICA) showed no significant changes in comparison with previous findings (Figure 1).

At this stage, the most beneficial reperfusion strategy should be followed. Although the common risk stratification models indicated an intermediate surgical risk (EuroSCORE II 1.2%, STS score 8.4%) and the SYNTAX scores favored CABG (SYNTAX score I was 23, SYNTAX score II was 39.4 for PCI with 4-year mortality of 14.4% and 21 for CABG with 4-year mortality of 3.3%), our patient's comorbidities made the actual surgical risk higher than that typically calculated. On the other hand, the implementation of a successful PCI was challenging with a high periprocedural risk, as coronary lesions were complex; they concerned, firstly, a chronic total occlusion (CTO) of the RCA and secondly, an acute-angled bifurcation lesion involving the LAD and a major diagonal branch (D1) with diffuse disease extending more than 15 mm from its ostium. Those bifurcation characteristics made the use of a complex 2-stent technique unavoidable.

The final joint decision of the Heart Team and the patient was the interventional approach, while the surgical option was kept as a minor alternative. Although computed tomography angiography could provide some more information, this was not feasible. Based on the ICA, the Japanese CTO score of the RCA lesion was calculated to be 0, as it would be the first attempt of PCI in a short-length CTO lesion with tapered entry and without calcification or bending within the segment. Our strategy involved beginning with the CTO PCI of the RCA due to its favorable anatomical features and in order to avoid any complications of the donor artery and afterwards, continuing with the PCI of the LAD/D1 bifurcation lesion.

**Figure 2:**
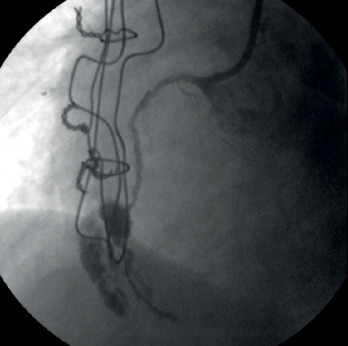
Ellis type III RCA perforation.

**Figure 3:**
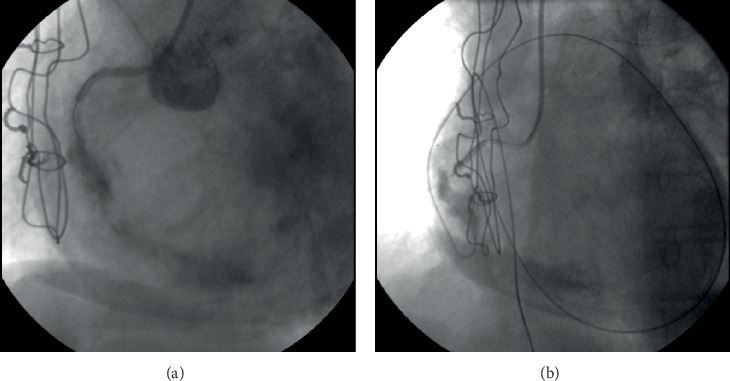
(a) Extravasation causing cardiac tamponade. (b) Urgent pericardiocentesis under fluoroscopic guidance.

**Figure 4:**
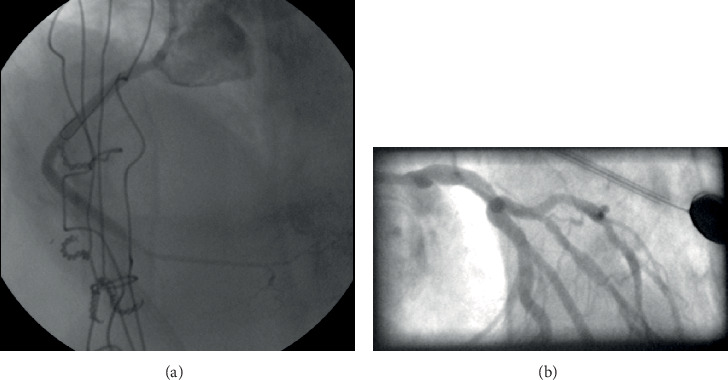
Final angiographic results. (a) CTO PCI of RCA; (b) PCI of LAD/D1 bifurcation lesion.

At the same time, another issue was the lack of a second arterial access, as there was a patent left radial artery, whereas the right radial artery was occluded following the previous ICA and the common femoral arteries were severely diseased (radiation-induced PAD). Patient's frailty and the fact that we wanted to avoid any complications of the brachial arteries made us proceed with a single left radial artery access using the antegrade wire escalation technique. The proximal lesion of the RCA was crossed, and a stiff guidewire was advanced to the distal vessel with a free wire movement. After having confirmed the position of the guidewire with two consecutive vertical views, a predilatation with a 1.5/15 mm semicompliant balloon was performed. Unfortunately, an Ellis type III perforation of the vessel was caused (Figure 2), as the balloon predilatation was found to have been implemented in an acute marginal branch that ran parallel to the main vessel [5]. Inflating a balloon proximally to the perforation was intended to stop further blood extravasation. Nevertheless, the patient soon became severely symptomatic with hemodynamic compromise because of rapid fluid accumulation into the pericardial cavity. Urgent pericardiocentesis under fluoroscopic guidance (Figure 3) fortunately stabilized the patient. Restoration of blood flow was finally achieved with the deployment of three drug-eluting stents after wiring the lumen of the RCA. The final angiographic result of the CTO PCI of the RCA was very good (TIMI flow grade 3) (Figure 4), and the patient was transferred to the coronary care unit being hemodynamically stable and asymptomatic.

A week later, the patient reentered the catheterization laboratory. The severity of the LAD lesion was assessed with fractional flow reserve (FFR) measurement that turned out to be 0.8. Double kissing crush technique using two stents was adopted due to the anatomical features of the bifurcation lesion that involved a large lumen diagonal branch with rich blood distribution arising at a steep angle from LAD (steep angle between D1 and LAD distal to the bifurcation).

An excellent angiographic result was finally succeeded (Figure 4), and 3 days later, the patient was discharged being asymptomatic. Eighteen months later, she still remains in the same good asymptomatic condition.

## 3. Discussion

The therapeutic strategy in patients with CAD and comorbidities is challenging and should be individualized based on the anatomical and functional characteristics of coronary arterial lesions, technical feasibility, and perioperative risk. Besides, after having been fully informed regarding revascularization alternatives, patient's will play the major role.

As mentioned above, our patient had plenty of comorbidities and even though the usual perioperative risk scores seemed to be intermediate and the SYNTAX score II indicated CABG for myocardial revascularization, there actually exist many features that are not taken into account by these risk models. ΜG and the history of myasthenic crisis experienced when operated with thymectomy, the prior sternotomy and thoracic radiation therapy creating uncertainty about the patency of the left internal mammary artery, and the long-term treatment with steroids discouraged essentially the option of CABG in our case.

MG is a rare disease caused by autoantibody-mediated blockade of the neuromuscular junction resulting in muscle disability [6]. Such patients are unpredictably resistant or sensitive to various anesthetic medications and may develop either a myasthenic crisis, which is an exacerbation of the disease, or a cholinergic crisis caused by cholinesterase inhibitor overdose [6]. Both conditions may need endotracheal intubation, while surgery may be by itself a triggering factor of myasthenic crisis frequently requiring immediate immunomodulating treatment [6].

There is an ongoing discussion regarding whether prior chest radiation exposure increases adverse outcomes of patients undergoing cardiac surgery. According to Wu et al., increased long-term mortality was observed in patients with prior thoracic radiation exposure treated with cardiac surgery [7]. In particular, the authors concluded with considering other treatment options as more appropriate in such cases. In contrast, Fender et al. demonstrated in their study that surgical revascularization in such patients could be performed as safely as in the control group [8]. Nevertheless, multiple studies conclude with the adverse effect of radiotherapy on internal mammary artery integrity and on its potential usage in CABG, which is a major factor of long-term survival [8, 9]. So, preoperative angiographic evaluation of the integrity of the graft may be considered.

Another field of discrepancy is whether repeat sternotomy portends a poorer prognosis. While some studies report that intraoperative injury can occur in as many as 9-9.1% of redo sternotomies, other authors report much lower risk [10–12]. Although redo sternotomies have been traditionally thought to carry higher mortality rate than first-time operations, this cannot be supported by all ongoing research and neither can its association with intraoperative injuries [10–13]. In either case, detailed preoperative planning including computed tomography scans may be very beneficial, along with a proper surgery technique such as hemisternotomy [12].

CR with complex PCI is a common practice nowadays and is associated with favorable outcomes concerning both morbidity and mortality. Farooq et al. showed that residual CAD with SYNTAX score greater than 8 following PCI was a determinant of 5-year mortality [14]. According to the 2018 guidelines of the European Society of Cardiology and the European Association for Cardio-Thoracic Surgery on myocardial revascularization, the choice of CABG instead of PCI should be guided by the potential for achieving CR [15]. Moreover, FAME and FAME 2 studies showed that the goal of CR via PCI should be preferably directed by functional characteristics and not by anatomical ones [15–17]. However, complex PCI procedures are associated with higher rates of complications; therefore, operators must be well prepared regarding not only the preprocedural planning but also the intervention itself in order to achieve a high quality result and deal with possible complications [4].

CTO PCI is a rapidly growing field of interventional cardiology that needs particular attention in safety issues. After obtaining dual contrast injection, several factors such as the position and the morphological features of the proximal and distal cap, the lesion length, the presence of branches, and retrograde techniques adequacy have to be assessed prior to the intervention [18]. However, PAD and the frailty of our patient made it hard to obtain a second arterial access that proved an unwise decision after all. Then, the similar course of RCA and acute marginal branches may mislead even an experienced operator. Imaging devices such as intravascular ultrasound may have beneficial contribution in proper wire crossing and balloon dilation. As regards complications, Patel et al. showed in their study that vessel perforation seemed to be the most common concern among cardiologists, whereas death and tamponade were their second and third most frequent single responses accordingly [19]. Consequently, urgent pericardiocentesis is a life-saving skill that all interventional cardiologists should be familiar with, while readily available pericardiocentesis kits in combination with either fluoroscopic or echocardiographic guidance may facilitate the procedure.

Last but not least, there comes the reliability of FFR in evaluating the severity of LAD lesions prior to and following the CTO PCI of RCA. Even though FFR in the culprit vessel is not reliable in the acute phase of ST-elevation myocardial infarction [20, 21], transient microvascular dysfunction is likely to be more limited in NSTEACS, so lesions in both the culprit and the nonculprit arteries may be reliably evaluated with FFR in those cases [20–22]. Then, as shown in Figure 1, the RCA territory is supplied by collaterals from the LAD. This results in larger total blood supply from the LAD compared to this vessel's supply in case of patent RCA. Regarding CTOs, the supply of the occluded-vessel territory depends on the patency and flow of the donor artery. When the last is affected by intermediate stenosis, FFR in the donor artery may normalize after successful PCI of the vessel with the CTO [23, 24]. Therefore, CTO recanalization followed by reassessment of the donor artery stenosis seems to be the proposed treatment plan in these cases [24].

In conclusion, although the common risk score models guiding the decision between PCI and CABG are helpful, in fact, they do not take into account various patient comorbidities. Individualization, case-by-case analysis, and patient's wishes are the key points of the therapeutic strategy. The evolution of PCI techniques, particularly in the field of CTO, makes interventional treatment possible in cases that were formerly managed exclusively with CABG. Nevertheless, this nonsurgical approach does not lack complications that every catheterization laboratory should be adequately trained to deal with.

## Abbreviations

CABG: Coronary artery bypass grafting
CAD: Coronary artery disease
CR: Complete revascularization
CTO: Chronic total occlusion
D1: First diagonal branch
FFR: Fractional flow reserve
ICA: Invasive coronary angiography
LAD: Left anterior descending
MG: Myasthenia gravis
NSTEACS: Non-ST elevation acute coronary syndrome
NSTEMI: Non-ST elevation myocardial infarction
PAD: Peripheral artery disease
PCI: Percutaneous coronary intervention
RCA: Right coronary artery.
